# Anatomical variations and congenital anomalies of the ribs revisited by multidetector computed tomography

**DOI:** 10.1590/0100-3984.2019.0131

**Published:** 2020

**Authors:** Lucas de Pádua Gomes de Farias, Daniel Cunha Menezes, Igor Schneider Faé, Pedro Henrique Coelho de Arruda, João Manoel Miranda Magalhães Santos, Gustavo Borges da Silva Teles

**Affiliations:** 1 UnitedHealth Group (UHG), São Paulo, SP, Brazil.

**Keywords:** Ribs, Anatomical variation, Multislice computed tomography, Arcos costais, Variantes anatômicas, Tomografia computadorizada multidetectores

## Abstract

As they are asymptomatic or have a nonspecific, anatomical variations of the ribs are usually detected as incidental findings on imaging studies. They may be isolated changes or can be related to anomalies or clinical syndromes. Such variations are easily overlooked on conventional radiography and computed tomography if they are not actively investigated, mainly because most indications for a chest X-ray studies aim to evaluate the lung parenchyma and mediastinal structures. The objective of this pictorial essay was to use multislice computed tomography images to illustrate the imaging aspects of the main anatomical variations and congenital anomalies of the ribs.

## Abstract (pt)

As variantes anatômicas dos arcos costais são, geralmente, achados incidentais nos exames de imagem, em razão do seu comportamento assintomático ou quadro inespecífico, podendo constituir alterações isoladas ou relacionadas a anomalias e síndromes clínicas. Essas alterações são facilmente negligenciadas na radiografia convencional e na tomografia computadorizada se não forem investigadas ativamente, principalmente quando a maioria das indicações do estudo radiológico do tórax tem como objetivo a avaliação do parênquima pulmonar e das estruturas mediastinais. O objetivo deste artigo é demonstrar, por meio da tomografia computadorizada multidetectores, os aspectos de imagem das principais variantes anatômicas e anomalias congênitas dos arcos costais.

## INTRODUCTION

Due to their asymptomatic or nonspecific presentation, anatomical variations of the ribs are typically discovered as incidental findings on imaging studies; they can be isolated changes or can be related to anomalies or clinical syndromes^(1,2)^. If they are not actively investigated, such variations are easily overlooked on conventional radiography and computed tomography^(1,2)^, especially because chest X-ray study is most often indicated for the assessment of the lung parenchyma and mediastinal structures.

The objective of this pictorial essay is to illustrate the imaging aspects of the main anatomical variations and congenital anomalies of the ribs. To that end, we employed images acquired with multislice computed tomography (MSCT).

## NORMAL ANATOMY OF THE RIBS

**Figure 1:**
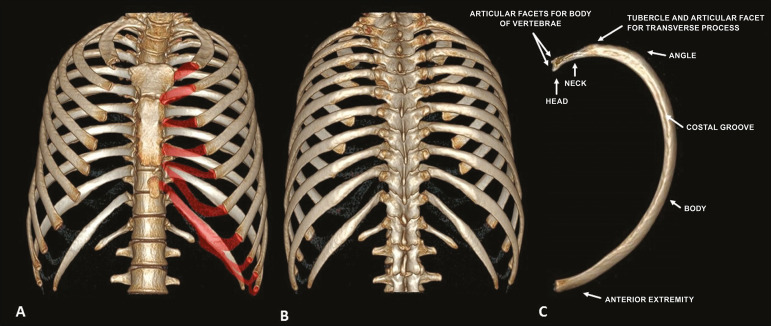
Anatomy of the ribs. MDCT (**A,B,C** - three-dimensional reconstructions) showing the usual anatomy of the ribs in a young adult characterized by 12 ribs articulated posteriorly to the respective vertebral bodies (T1 to T12). The first to seventh ribs (the true ribs) articulate directly to the sternum through the costal cartilages, whereas the eighth to the tenth ribs (false ribs) articulate indirectly to the sternum through the superior costal cartilage of the seventh ipsilateral rib and the eleventh and twelfth ribs (floating ribs) are points of origin and insertion of the myotendinous junction, without connecting to the sternum. Note the anatomical components of the rib, through its lower view (in **C**), and the projection of the hyaline costal cartilages (red transparency).

The thoracic cavity (Figure 1) consists of 12 pairs of ribs, the sternum, the clavicles and the thoracic segment of the spine. The ribs are flat, elastic bones that articulate anteriorly to the sternum and posteriorly to the vertebral bodies; they can be classified as true, false, or floating-according to their sternal articulation-or as typical or atypical-according to their morphology^(3,4)^.

The ribs are classified as true when they articulate directly to the sternum through their respective costal cartilages (synchondrosis in the first rib and synovial from the second to the seventh), false when they articulate indirectly to the sternum through the adjacent superior costal cartilage (synchondrosis), and floating when there is no anterior costal joint (i.e., when they end at the abdominal wall). The first seven ribs are true, the eighth to the tenth are floating, and the last two are false^(3-5)^.

From a morphologic perspective, the third to the tenth ribs are classified as typical because they have articular facets, with an elongated body connected to the rib head by the neck and a tubercle (Figure 1C). The head articulates with the vertebral body, forming the costovertebral joint, and the tubercle articulates with the transverse process of the vertebra, forming the costotransverse joint, both being synovial joints. The first, second, eleventh, and twelfth ribs are considered atypical because they do not have characteristics in common to the other ribs. The first and second ribs have characteristics of the cervicothoracic junction. The first rib is shorter and wider, with grooves related to the subclavian vessels and a tubercle for fixing the scalene muscles, whereas the second rib, thinner and longer than the first, has a tuberosity for fixing the anterior serratus muscle. The eleventh and twelfth ribs are atypical because they do not have anterior articular facets, a neck, or a tubercle. Every rib has a lower costal groove, related to the intercostal neurovascular bundle; the second to the ninth ribs have a second articular face on their head for articulation with the adjacent upper vertebral body^(3,4)^.

## PHYSIOLOGICAL COSTOCHONDRAL CALCIFICATIONS AND PSEUDARTHROSIS OF THE FIRST RIB

**Figure 2:**
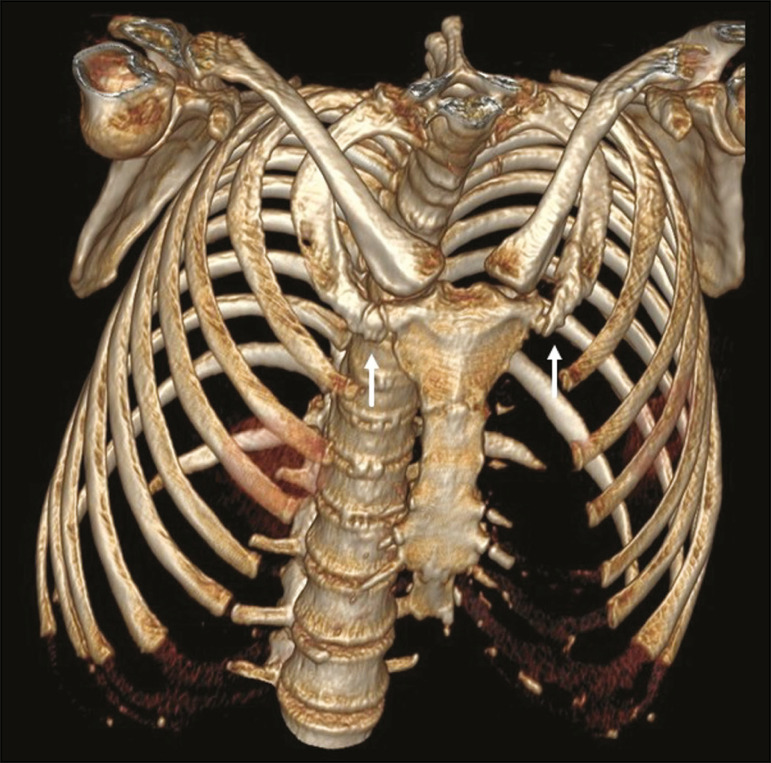
Pseudarthrosis and physiological costochondral calcifications. MDCT (three-dimensional reconstruction) showing pseudarthrosis in the first bilateral rib (arrows) in a 45-year-old female patient. Note the pattern of nodular and peripheral calcification of bilateral sternocostal cartilage.

Costochondral cartilages typically present calcifications after the end of the second or beginning of the third decades of life; when seen in adolescents or young adults, such calcifications can be associated with pathological conditions such as renal failure, autoimmune diseases, thyroid diseases, and neoplasms^(4)^. As the cartilaginous calcification of the first rib progresses (Figure 2), pseudarthrosis becomes a quite common finding^(3,6)^. The MDCT pattern of calcification differs between female and male patients (Figures 2 ands 3, respectively): women tend to have central and nodular calcifications, with a tongue aspect, whereas men tend to have parallel peripheral linear calcifications, with a tram-track aspect^(4,5)^.

**Figure 3:**
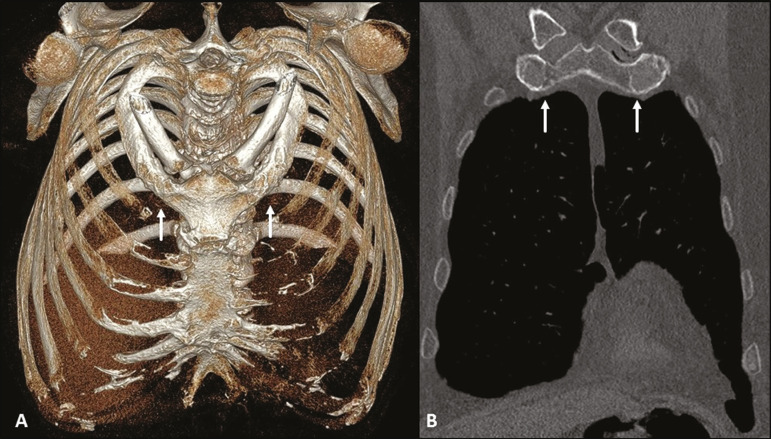
Physiological costochondral calcifications. MDCT (**A** - three-dimensional reconstruction; **B** - coronal view) showing fusion of the first rib to the sternal manubrium (arrows) in an 83-year-old male patient, together with calcification of the other sternocostal and bilateral chondrocostal cartilages. Note the tramtrack pattern of calcification.

## CERVICAL RIB

**Figure 4:**
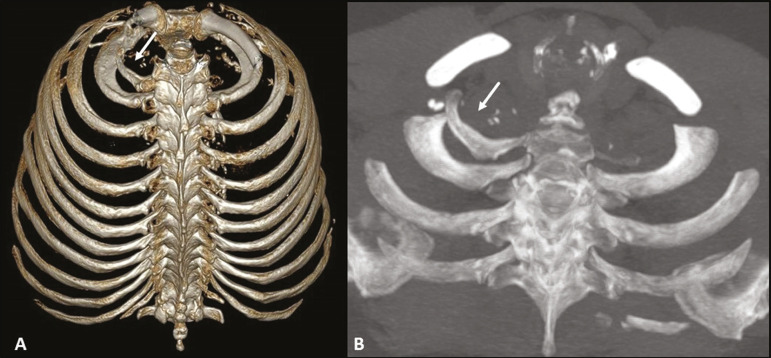
Cervical rib. MDCT (**A** - three-dimensional reconstruction; **B** - axial maximum intensity projection) showing a supernumerary rib on the right (arrows), articulating posteriorly with the C7 vertebral body, with no articular structure in its aspect anterior to the sternum or any bony structure (floating ). Note its close relationship with the first thoracic rib and the ipsilateral clavicle, which can be related, in some cases, to vascular compression syndrome.

The cervical rib is an accessory or supernumerary rib that articulates posteriorly with the seventh cervical vertebral body (C7), as shown in Figure 4. Its prevalence in the general population ranges from 0.2% to 2%, and it is more common in females. However, it is a common finding in individuals with Klippel-Feil syndrome. It can be unilateral or asymmetrically bilateral and is typically asymptomatic. It can be related to brachial plexus neuropathy and vascular compressions, such as thoracic outlet syndrome and aneurysms of the ipsilateral subclavian artery. The differential diagnosis should include an elongated transverse process of C7 or a short first rib^(1,2,7,8)^.

## SHORT RIB

**Figure 5:**
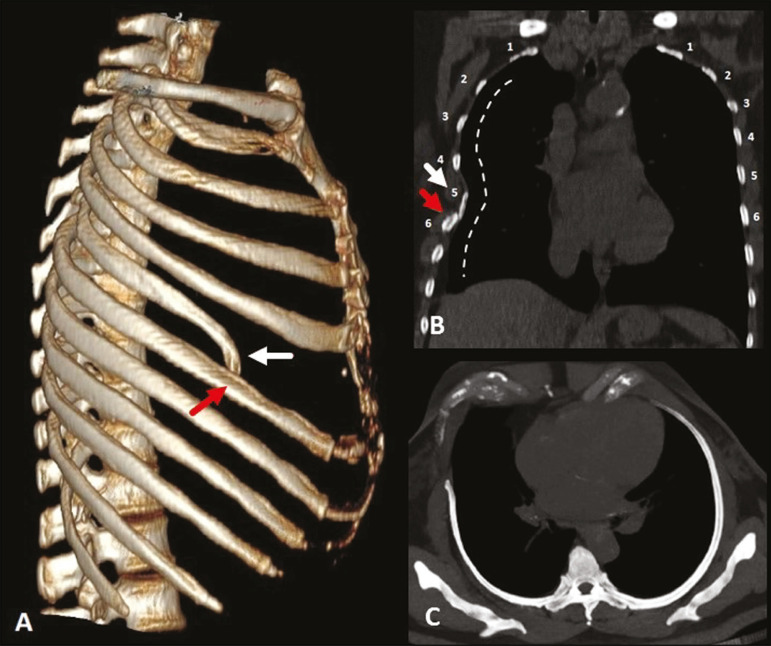
Short rib. MDCT (**A** - threedimensional reconstruction; **B** - coronal view; **C** - axial oblique maximum intensity projection on the longitudinal axis of the rib) showing hypoplasia of the right fifth rib that presents a lower inclination at its anterior end (white arrows), juxtaposed with the middle aspect of the ipsilateral sixth rib (red arrows). Note the asymmetry when compared with the contralateral rib in the coronal and axial images, together with the locoregional deformity of the rib cage (dotted line).

A short (hypoplastic) rib is one that does not extend anteriorly as far as the sternum (Figure 5), probably due to early fusion of the epiphyseal growth plate^(2,7)^. This variation occurs in approximately 16% of the population, is more common on the right than on the left, and can occur bilaterally (7). It is usually asymptomatic, constituting an isolated finding, but may be associated with skeletal dysplasias, such as thanatophoric dysplasia, achondroplasia, Ellis-van Creveld syndrome (chondroectodermal dysplasia), Jeune syndrome (asphyxiating thoracic dystrophy), and other short-rib polydactyly syndromes^(2)^.

## BIFID RIB

**Figure 6:**
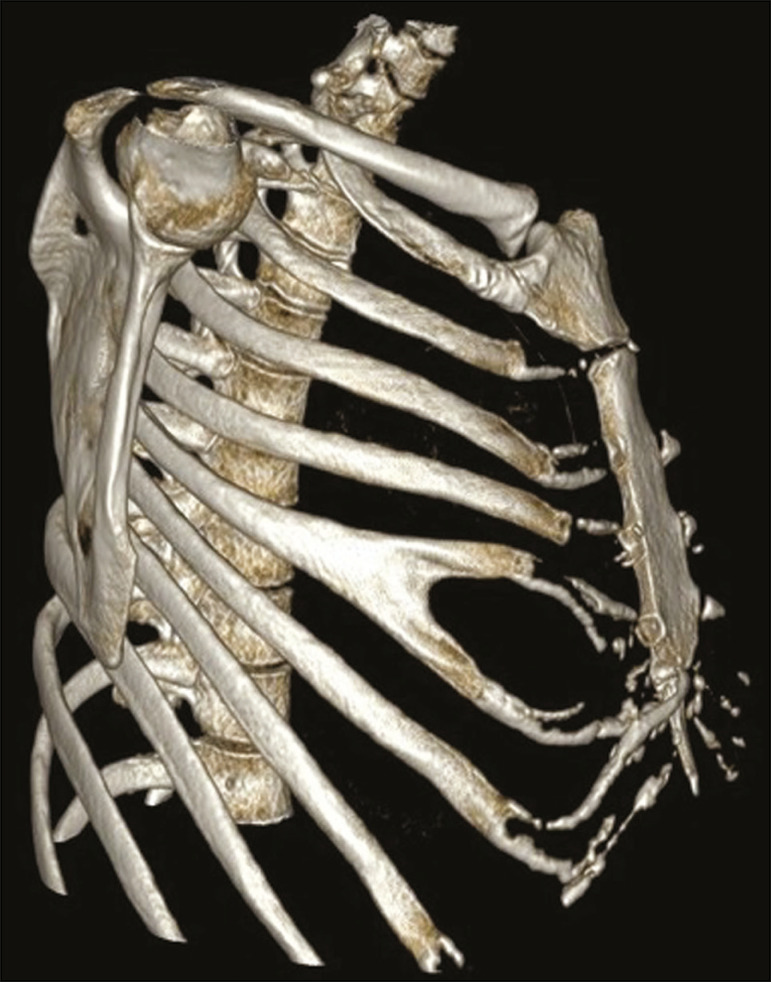
Bifid rib. MDCT (three-dimensional reconstruction) showing the bifid aspect of the right fifth rib in its anterior portion, including the sternocostal hyaline cartilage, although without causing significant deformities in the rib cage. Bilateral sternocostal and chondrocostal calcifications.

Also known as Luschka’s forked rib (Figure 6), a bifid rib is the most common variation, characterized by a division in its anterior portion, in its bony and cartilaginous aspects, typically affecting the fourth rib^(1,2)^. It is also associated with Gorlin syndrome^(2)^.

## INTRATHORACIC RIB

**Figure 7:**
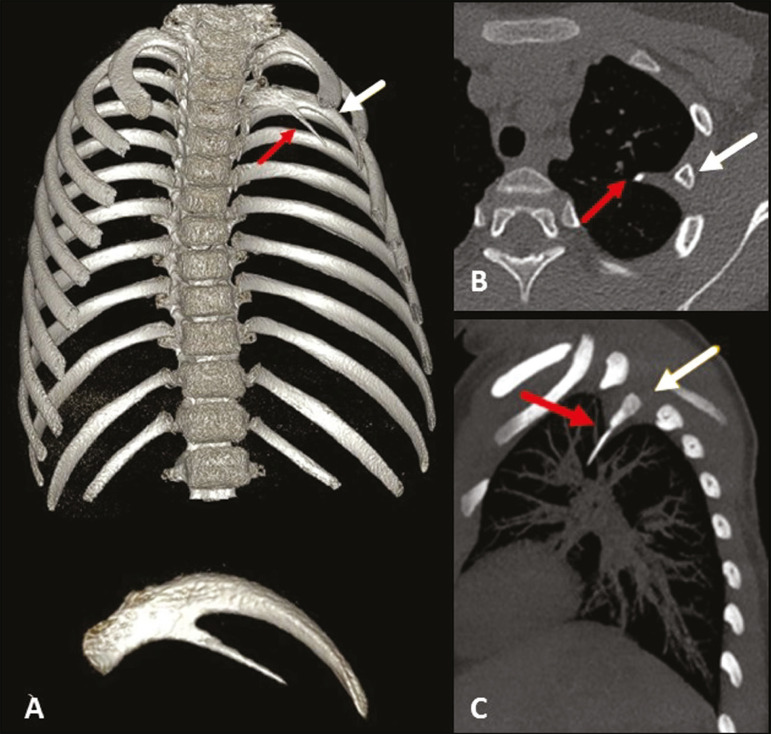
Intrathoracic rib (type Ib). MDCT (**A** - three-dimensional reconstruction; **B** - axial view; **C** - sagittal maximum intensity projection) showing a supernumerary rib (red arrows) originating in the posterior portion of a short left third rib (white arrows), with a lateral, inferior oblique path to the pulmonary parenchyma. Note that the rib has no visible medullary cavity. No changes were identified in the posterior vertebral bodies or ribs.

An intrathoracic rib is a very rare variation, characterized by a bony prominence of a rib into the chest cavity^(9,10)^. It is usually a supernumerary rib, unilateral (most commonly on the right), and asymptomatic, although it may have diaphragmatic insertion with repercussions on respiratory function^(10,11)^. There is a proposed classification related to genetic changes^(9)^: type Ia = supernumerary rib originating from the anterolateral portion of the vertebral body; type Ib = supernumerary rib originating from the posterior portion of another rib, adjacent to the vertebral body (Figure 7); type II = rarer, bifid rib with the intrathoracic bony segment in the distal portion of the rib; and type III = depression of the rib into the chest cavity. The association of more than one type is also possible.

## RIB FUSION

**Figure 8:**
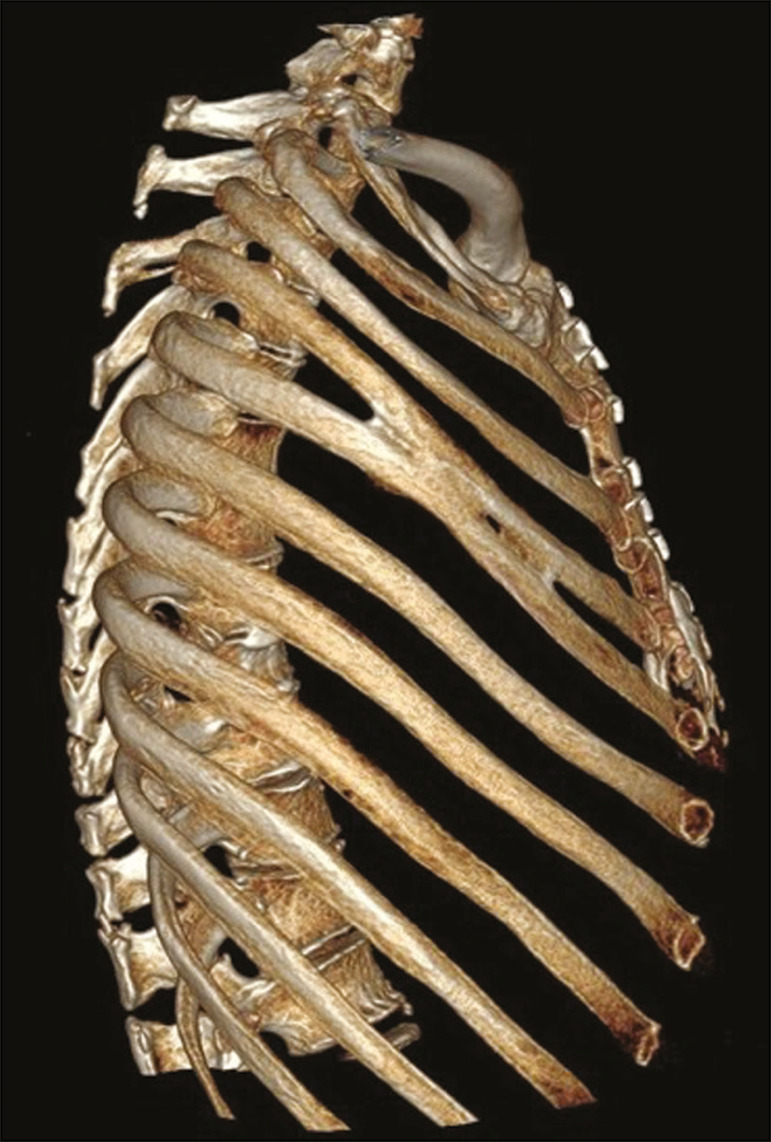
Rib fusion. MDCT (three-dimensional reconstruction) showing fusion of the middle body portions of the fourth and fifth ribs on the right, although without causing significant deformities in the rib cage.

Rib fusion can be complete or partial, affecting the anterior portion of the rib (Figure 8) or its posterior portion. It is believed to result from a failure of segmentation, because it can also be related to segmentation failure of vertebral bodies^(1)^.

## RIB FORAMEN

**Figure 9:**
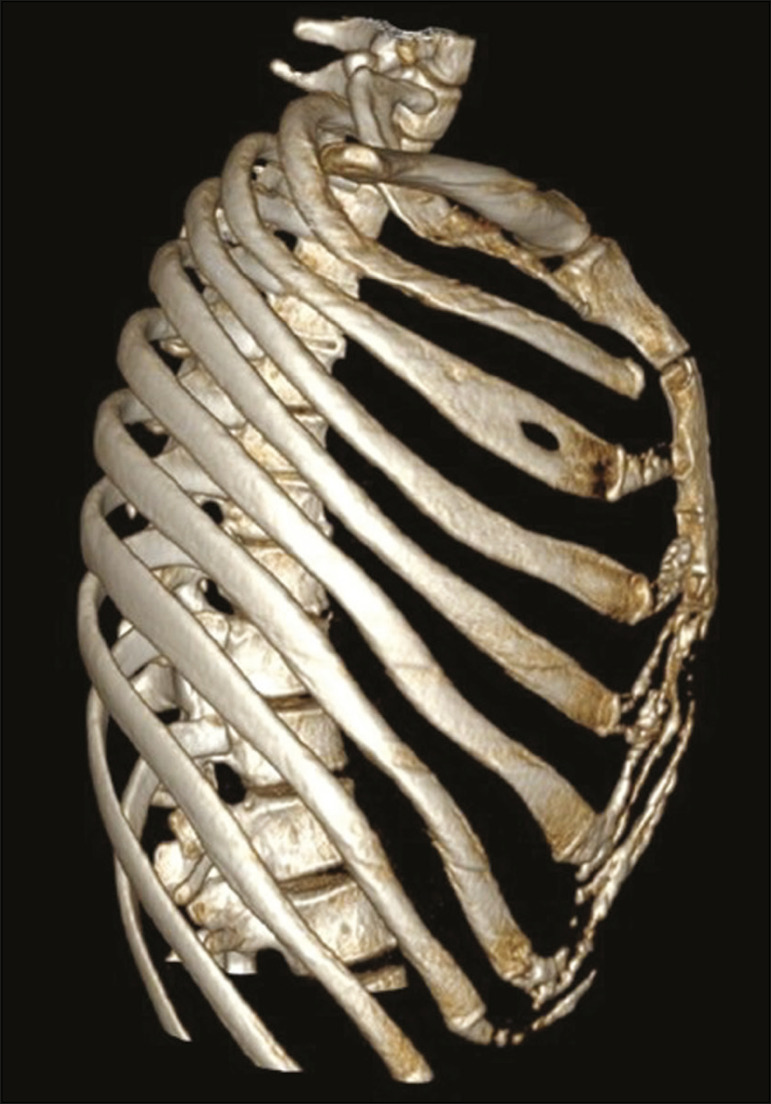
Rib foramen. MDCT (three-dimensional reconstruction) showing a well-circumscribed foramen, with corticated contours, in the anterior region of the right third rib.

On MDCT, a rib foramen presents as a rounded image of a well-corticated defect centered in the bony component of the rib (Figure 9). Although it is not clinically significant, rib foramen should be included in the differential diagnosis of bone lesions, together with aneurysmal bone cyst and enchondroma^(12)^.

## RIB NOTCHING

**Figure 10:**
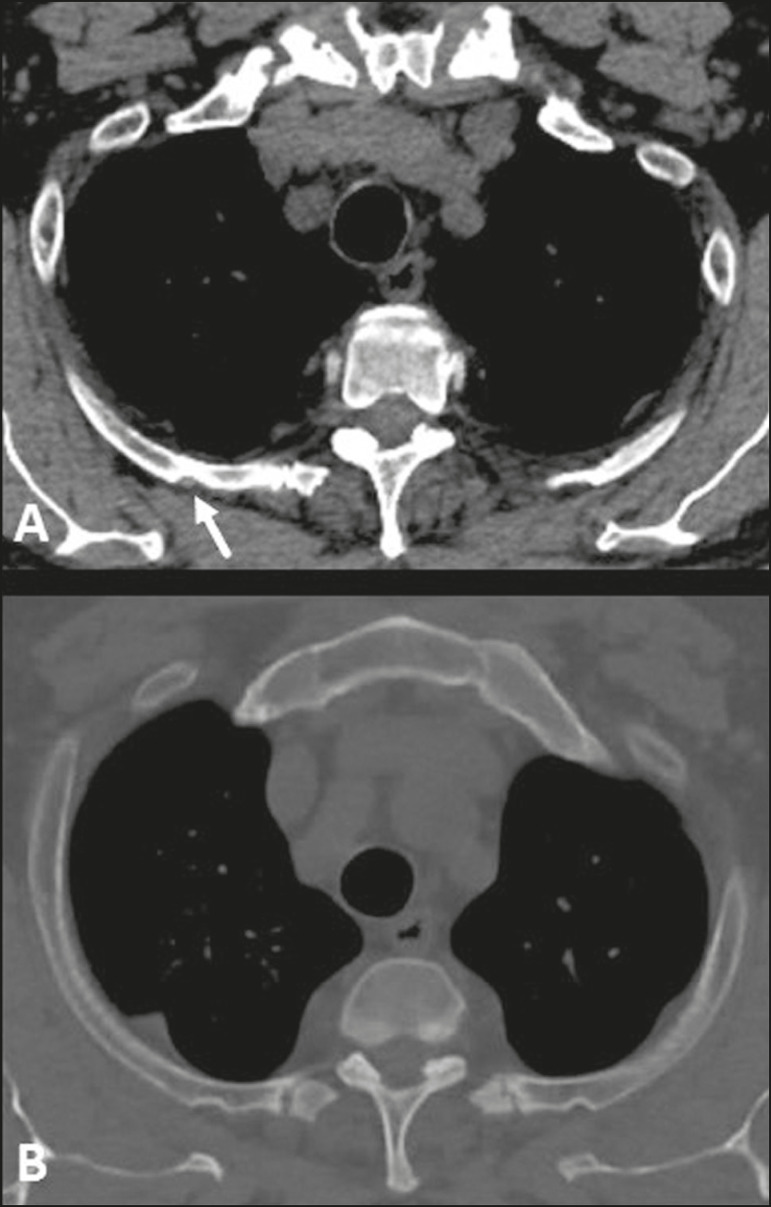
Rib notching. MDCT (A,B - axial oblique view) showing bone deformation in the posterior aspects of the ribs on both sides, most evident in the upper ribs on the right, which follow the arterial vascular path (arrow).

Rib notching are deformities that affect the upper surface of the rib, the lower surface of the rib (Roesler’s sign), or both. They can be related to arterial (Figure 10), venous, neurogenic or connective tissue diseases, or other changes that cause an increased local pressure on the rib^(2)^, such as the prominent vascularization of the inferior costal groove in some cases of coarctation of the aorta^(13)^.

## CONCLUSION

The ribs present numerous normal radiological aspects, anatomical variations, and pathological conditions, sometimes mimicking alterations of the lung parenchyma on radiography, which are best elucidated by MSCT. Their accurate evaluation is extremely important, because various imaging findings can be useful as indicators of known or unknown bone dysplasia, heart disease, metabolic disease, trauma, and neoplasia. Radiologists should be familiar with the variations from normality, in order to avoid confusing them with pathological conditions.
